# Humoral Response to BNT162b2 Vaccine Against SARS-CoV-2 Variants Decays After Six Months

**DOI:** 10.3389/fimmu.2022.879036

**Published:** 2022-05-02

**Authors:** Tulio J. Lopera, Mateo Chvatal-Medina, Lizdany Flórez-Álvarez, Maria I. Zapata-Cardona, Natalia A. Taborda, Maria T. Rugeles, Juan C. Hernandez

**Affiliations:** ^1^ Grupo Inmunovirología, Facultad de Medicina, Universidad de Antioquia, Medellín, Colombia; ^2^ Grupo de Investigaciones Biomédicas Uniremington, Programa de Medicina, Facultad de Ciencias de la Salud, Corporación Universitaria Remington, Medellín, Colombia; ^3^ Infettare, Facultad de Medicina, Universidad Cooperativa de Colombia, Medellín, Colombia

**Keywords:** SARS-CoV-2, vaccine, neutralizing antibodies, immunity, immunogenicity, variant

## Abstract

SARS-CoV-2 vaccines have shown very high effectiveness in real-world scenarios. However, there is compelling evidence for a fast-paced waning of immunity. The increasing number of new variants that could alter the severity, transmissibility, and potential to evade the immune response raised significant concern. Therefore, elucidating changes in the humoral immune response against viral variants induced by vaccines over time is crucial for improving immunization protocols. We carried out a 6-month longitudinal prospective study in which 60 individuals between 21 and 71 years of age who have received the complete scheme of the BNT162b2 vaccine were followed to determine titers of serum neutralizing activity. The neutralizing capacity was measured at one, three, and six-months post-vaccination by plaque reduction neutralization assay using SARS-CoV-2 B.1 (D614G) and the Gamma, Alpha, Delta, and Mu variants. Data were analyzed using GraphPad 5.0. Neutralizing activity against five different SARS-CoV-2 variants was detected in the serum samples of all vaccinated participants to a different extent after one month, with a progressive decrease according to age and gender. Overall, after one month of vaccination, the neutralizing titer was lower for all evaluated variants when compared to B.1, most remarkable against Delta and Mu, with a reduction of 83.1% and 92.3%, respectively. In addition, the Titer at 3- or 6-months follow-up decreased dramatically for all variants. Our results support the decaying of serum neutralizing activity, both over time and across SARS-CoV-2 variants, being more significant in older men. Since Delta and Mu appear to evade the neutralizing activity, these and further new variants of immune escape mutations should be considered for novel vaccine formulations.

## Introduction

A few times throughout its history, humanity has faced such significant threats as the SARS-CoV-2 pandemic. The appearance of this novel coronavirus has resulted in substantial morbidity and mortality worldwide, which in turn has precipitated a vast spectrum of pharmacologic and non-pharmacologic measures to contain its impact (1). Some of the most encouraging ones include vaccines that provide immunity against symptomatic infection, reducing transmission to a variable extent (2, 3). Although overall vaccine effectiveness has proven to be very high in real-world scenarios, there is compelling evidence for a fast-paced waning of immunity. Some studies reported initial effectiveness of nearly 90% during the first month, which declined to under 50% after five months (4). A further concern has been raised with the increasing number of new variants with distinct mutations that might increase disease severity, transmissibility, and immune evasion, which might render a significant population vulnerable, either formerly immunized or not (5).

SARS-CoV-2 has undoubtedly reshaped the understanding of viral evolution. Despite its proven mechanisms for maintaining genetic fidelity, biological pressure has led to selecting highly fit specimens that are now considered variants of interest (VOIs) or variants of concern (VOCs) (6). The World Health Organization (WHO) has currently designated five VOCs -Alpha, Beta, Gamma, Delta, and Omicron- and two VOIs -Lambda and Mu-, many of which contain important mutations for immune evasion such as E484K and N501Y (5, 7). Since immunity from vaccines could exert selective pressure on viral evolution and given the possibility of potential escape from antibody neutralization by these variants, a particular emphasis must be set on how vaccine effectiveness is affected as they continue being rolled out worldwide. In fact, worrying data have already surfaced, showcasing a decrease in effectiveness against variants such as Delta, where overall vaccine effectiveness against infection varies from 51.9 to 88% (8, 9).

Extensive research has been conducted on the kinetics of immune response against this virus, both from natural infection and vaccines (10, 11). Nonetheless, there is little evidence so far regarding the behavior of such responses against multiple variants lengthwise, and this holds especially true for recently characterized variants such as Mu and Delta. Furthermore, a well-standardized study focusing on live-virus neutralization is peremptory, as these assays have allowed for some correlates of protection (12, 13). This longitudinal study aimed to evaluate the overall and variant-specific immunogenicity through live-virus neutralization of BNT162b2 against Alpha, Gamma, Delta, Mu, and B.1 SARS-CoV-2 variants in a group of individuals in Colombia.

## Materials and Methods

### Study Design and Volunteers

We conducted a prospective longitudinal cohort study in Medellin, Colombia, between May and November of 2021, in BNT162b2 fully vaccinated individuals (Pfizer - BioNTech). The study was designed and conducted following the Declaration of Helsinki and Colombian legislation (Ministry of Health resolution 008430 de 1993). It was approved by the Ethics Committee of the Universidad de Antioquia (Acta 006/2021). After thoroughly explaining the project, all subjects signed a written informed consent and provided blood samples.

The study population included healthcare professionals or individuals prioritized in the early stages of vaccination in Colombia. All individuals received the BNT162b2 vaccine in a double-dose scheme, with an inter-dose interval of 3 weeks, as per the interim recommendations issued by the WHO. Eligibility criteria included an age of 18 years or older and a complete BNT162b2 vaccination schedule. In total, 60 people were included, and they were classified into four groups according to gender and age (women and men under and over 40 years).

Exclusion criteria included any history of SARS-CoV-2 infection prior to the first dose (defined as any spectrum of confirmed infection or symptoms suggestive of COVID-19), suspected SARS-CoV-2 infection at the time of inclusion in the study, incomplete schedule, or completed schedule vaccination more than 35 days ago, people in pregnancy, with autoimmune diseases, cancer, or HIV-1 infection. In the event of any symptoms associated with COVID-19 or exposure to a person infected with SARS-CoV-2, a RT-qPCR test for SARS-CoV-2 was performed. Those individuals with confirmed SARS-CoV-2 infection or who received the third vaccination dose in the follow-up period were excluded (n=4).

### Follow-Up of Vaccinated Individuals

The individuals were followed for 180 days from a complete vaccination schedule. All volunteers provided a peripheral-blood sample at 30, 90, and 180 days after receiving the second vaccination dose (with a window of ± five days on days 30 and 90, and ± 28 days on day 180). At the beginning of the study, each individual completed a survey on demographic information, report of symptoms associated with vaccination, and existence of comorbidities. After the first visit, participants were followed with a virtual survey where they were asked about the onset of symptoms suggestive of COVID-19, confirmed infection by SARS-CoV-2, or the diagnosis of new comorbidities.

### Plaque Reduction Neutralizing Test

Neutralizing activity of serum samples was detected by a 50% plaque reduction neutralization test (PRNT50) using Vero E6 cells. Briefly, Vero E6 cells (1.1 x 10^5^ cells per well) were seeded into the 24-well tissue culture plates. The next day, 100 plaque-forming units (PFU) of SARS-CoV-2 were incubated with serial dilutions (1:20 until 1:5120) of heat-inactivated serum samples (56°C, 30 min) in a final volume of 500 μL for 60 min at 37°C and 5% CO2. Then, the mix was added to the Vero E6 monolayers by duplicate (200 uL per well) and incubated at 37°C for 60 min. Subsequently, the inoculum was removed, and 1 ml of the semisolid medium (1.5% carboxymethylcellulose, 2% fetal bovine serum (Gibco, Grand Island, NY, USA), 1% streptomycin (Sigma-Aldrich, St. Louis, MO, USA), and DMEM (Dulbecco’s Modified Eagle Medium, Sigma-Aldrich, St. Louis, MO, USA) was added and incubated at 37°C for 72h. Then, the semisolid medium was removed, and the monolayers were washed twice with PBS (Lonza, Rockland, ME, USA). Finally, the monolayers were fixed and stained with 1% crystal violet and 4% formaldehyde for 30 min and washed twice with PBS. A 50% reduction in plaque count was defined as the neutralization endpoint. The percentage inhibition was calculated based on the number of plaques in the infection control wells. The higher dilution with a reduction of 50% of plaques was reported as plaque reduction neutralization titer (PRNTi)

Neutralizing activity was evaluated in all participants using5 different lineages obtained from viral isolates collected in Colombia. The lineages assessed were B.1 (D614G) (hCoV-19/Colombia/ANTUdeA-200325-01/2020 ID accession: EPI_ISL_536399), variants of concern (VOC) Gamma (P.1) (hCoV-19/Colombia/ANT-UdeA-21002835v/2021 ID accession: EPI_ISL_4926393), Alpha (B.1.117) (hCoV-19/Colombia/ANT-UdeA-21001965v/2021 ID accession: and Delta (B.1.617.2) (hCoV-19/Colombia/ANT-UdeA-36211/2021 ID accession: EPI_ISL_5103929), and the variant of interest (VOI) Mu (B.1.621) (hCoV-19/Colombia/ANT-UdeA-21002149/2021 ID accession: EPI_ISL_4005445). Colombia/ANT-UdeA-21002149/2021 ID accession: EPI_ISL_4005445), and the variant of interest (VOI) Mu (B.1.621) (hCoV-19/Colombia/ANT-UdeA-36211/2021 ID accession: EPI_ISL_5103929).

### Statistical Analysis

We performed an exploratory analysis to identify atypical data. The distribution of demographic, clinical, and immunological variables was assessed using the Shapiro-Wilk test. Wilcoxon test for paired samples and Spearman’s rank correlation coefficient were used to determine significant differences and correlations. All data were analyzed using GraphPad Prism software, version 8.0 (California, USA), and the significance statistic was defined as p-value <0.05.

## Results

### Sociodemographic Characteristics of Participants in the Study

Three serial serum samples have been taken from each individual up to the cut-off date, and their demographic characteristics are shown in **Table 1**. The cohort was evenly distributed among the groups of males and females, younger or older than 40. The mean body-mass index (BMI) was 24.8 Kg/^m2^, and 16.6% of individuals reported comorbidities. Arterial hypertension and diabetes were the most prevalent (5% and 3.33%, respectively). Over 70% of participants in each group presented local symptoms, and over 60% presented systemic symptoms related to vaccination, except in males over 40 years, where these reactions were noticeably lower (33.3%). Concerning profession, 41.7% of the study participants were either healthcare professionals or had increased exposure to the virus and were consequently prioritized for receiving the vaccine. After six months of follow-up, 176 samples were included in the study: after 30 days (n=60), 90 days (n=60), and 180 days (n=56) (4 donors were excluded because of SARS-CoV-2 infection during the follow-up).

**Table 1:** Demographic characteristics of participants fully vaccinated with BNT162b2.

		Female under 40 years old (n=15)	Male under 40 years old (n=15)	Female over 40 years old (n=15)	Male over 40 years old (n=15)
**Age: years [range]**		29 [21-39]	27 [21-40]	57 [47-68]	55 [42-71]
**Body-mass index [range]**		24.4 [18.0-28.9]	24.1 [19.5-31.4]	24.6 [21.1-29.3]	25.9 [23.1-30.9]
**Blood type**	**A (%)**	3 (20)	4 (26.7)	3 (20)	10 (66.7)
	**B (%)**	1 (6.7)	4 (26.7)	1 (6.7)	0 (0)
	**O (%)**	11 (73.3)	7 (46.7)	10 (66.7)	4 (26.7)
	**AB (%)**	0 (0)	0 (0)	1 (6.7)	0 (0)
**Rhesus factor**	**Positive (%)**	14 (93.3)	14 (93.3)	14 (93.3)	11 (73.3)
**Symptoms associated with vaccination**	**Local (%)**	12 (80)	13 (86.7)	13 (86.7)	7 (46.7)
	**Systemic (%)**	11 (73.3)	11 (73.3)	10 (66.7)	5 (33.3)
**Substance consumption**	**Alcohol (%)**	0 (0)	2 (13.3)	1 (6.7)	1 (6.7)
	**Cigarette (%)**	2 (13.3)	0 (0)	0 (0)	0 (0)
	**Other (%)**	0 (0)	0 (0)	0 (0)	0 (0)
**Exercise**	**Yes (%)**	9 (60)	13 (86.7)	8 (53.3)	7 (46.7)
	**No (%)**	6 (40)	2 (13.3)	8 (53.3)	10 (66.7)
	**Hours per week [range]**	3.5 [2-7]	4 [3-12]	4.5 [2-7]	5 [3-14]
**Comorbidities**	**Arterial hypertension (%)**	0 (0)	0 (0)	3 (20)	0 (0)
	**Cardiovascular disease (%)**	0 (0)	0 (0)	0 (0)	1 (6.7)
	**Diabetes (%)**	0 (0)	0 (0)	1 (6.7)	1 (6.7)
	Other* (%)	1 (6.7)	0 (0)	3 (20)	0 (0)

*Other comorbidities: Dyslipidemia, Gilbert’s syndrome, glaucoma, and hypothyroidism.

### Immunogenicity of BNT162b2 at Day 30 Post-Vaccination

**Figure 1:**
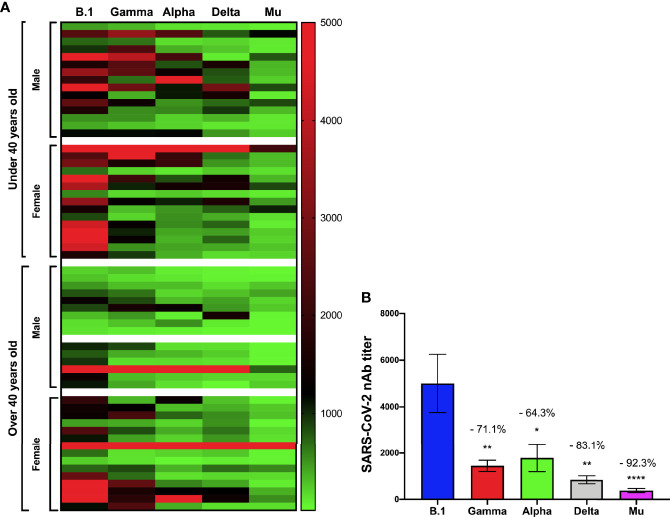
Immunogenicity of BNT162b2 at 30 days after being fully vaccinated. The neutralizing antibody (nAb) titers against the B.1, Gamma, Alpha, Delta, and Mu variants, 30 days after being fully vaccinated according to age over or under 40 years and gender is shown in **(A)**. The percentage decrease in the neutralizing titers against the Gamma, Alpha, Delta, and Mu variants compared to B.1 lineage is shown in **(B)**. *p<0.05. **p<0.01. ****p<0.0001.

PRNTi was detected in all serum samples after 30 days of completing the vaccination schedule. Participants younger than 40 years old showed a more robust response after vaccination, with PRNTi against B.1 higher than 320 (reciprocal dilution) in most cases (**Figure 1A**). The neutralization capacity was higher in female participants than in their male counterparts, as they more often reached titers of up to 5120. The PRNTi generated after vaccination were enormously effective against the B.1 virus, but were hindered against VOCs and the VOI. In particular, VOCs Gamma and Alpha showed a reduction in PRNTi titers of 71.1% and 64.3%, respectively, when compared with those against B.1. Even more so, VOC Delta and VOI Mu showed a profound reduction in neutralizing capacity, as PNRTi decreased by up to 83.1% with and 92.3% respectively (**Figure 1B**) when compared with B.1.

### Decreasing PRNTi Against All Variants in Older Individuals

**Figure 2:**
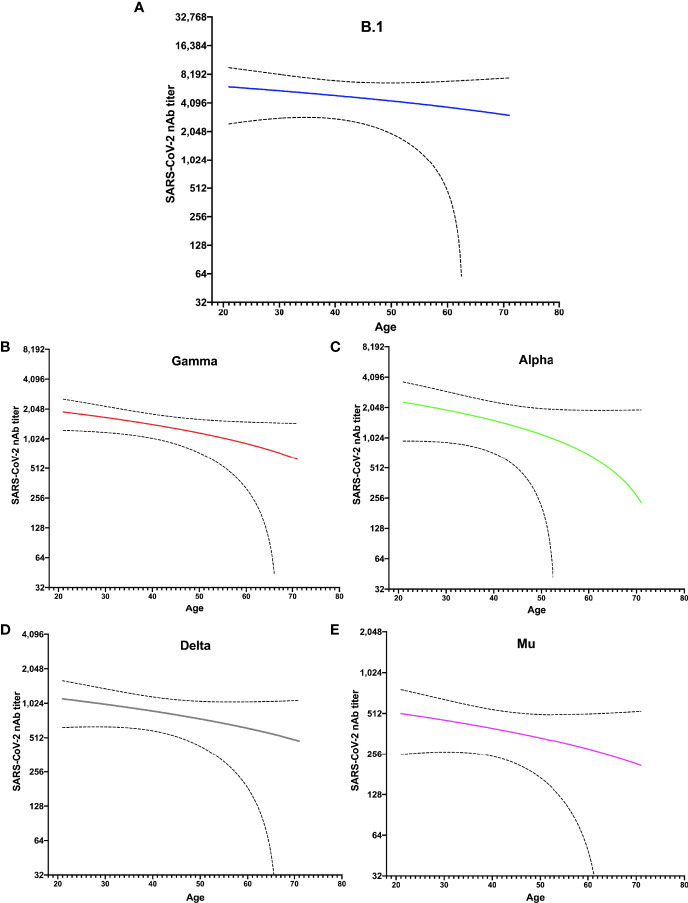
Lower nAbs titer against five variants studied in older donors. The distribution of neutralizing antibody (nAbs) titers after 30 days of completing the vaccination scheme is shown for each variant, according to the age distribution of studied individuals: **(A)** B.1 lineage, **(B)** Gamma, **(C)** Alpha, **(D)** Delta, and **(E)** Mu. The statistical analysis was performed using Spearman’s correlation.

To assess the difference among participants under and over 40 years old and the dramatic decrease in titers seen in all individuals against the variants, we analyzed SARS-CoV-2 PRNTis against each VOC and the VOI according to age. Older volunteers showed significantly lower PRNTi against all variants (**Figure 2**). Although a decrease in neutralization activity was observed in the entire population against the Gamma, Alpha, Delta, and Mu variants, compared with B.1, older individuals had lower PRNTi titers overall. We observed a significant decrease in neutralizing against the B.1 (r= -0.35, p= 0.0037), Gamma (r= -0.33, p= 0.0055), Alpha (r= -0.49, p< 0.0001), Delta (r= -0.37, p= 0.0017), and Mu (r= -0.39, p= 0.0011) (**Figure 2**).

### Neutralizing Capacity Against All Lineages Decays Over Time

**Figure 3:**
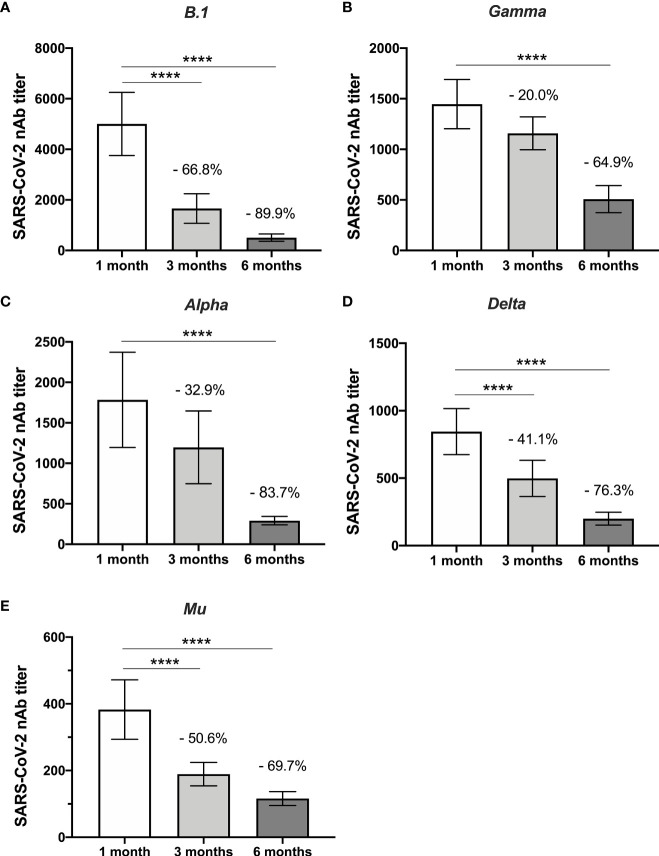
Decrease in nAbs against five variants studied over time. The comparison between the neutralizing antibody titers of the individuals after one month and three months of being fully vaccinated are shown according to the variants: **(A)** B.1 lineage, **(B)** Gamma, **(C)** Alpha, **(D)** Delta, and **(E)** Mu. The statistical analysis was performed using Wilcoxon’s test. ****p<0.0001.

Plaque reduction neutralizing titers showed an evident decrease over time since vaccination. A noticeable reduction in neutralizing activity between 1- and 6-months post-vaccination among all variants evaluated was observed. For B.1 SARS-CoV-2, a 20% decrease at 90 days and 64.9% at 180 days post-vaccination were observed. For the variants Gamma, Alpha, Delta, and Mu, a progressive decay rate of neutralizing titer was observed during the follow-up, reducing at six months post-vaccination of 89.9%, 83.4%, 76.1%, and 68.9%, respectively. This difference was statistically significant for Gamma, Delta, and Mu variants at three- and six months post-vaccination and for B.1 and Alpha only at six months post-vaccination (**Figure 3**).

**Figure 4:**
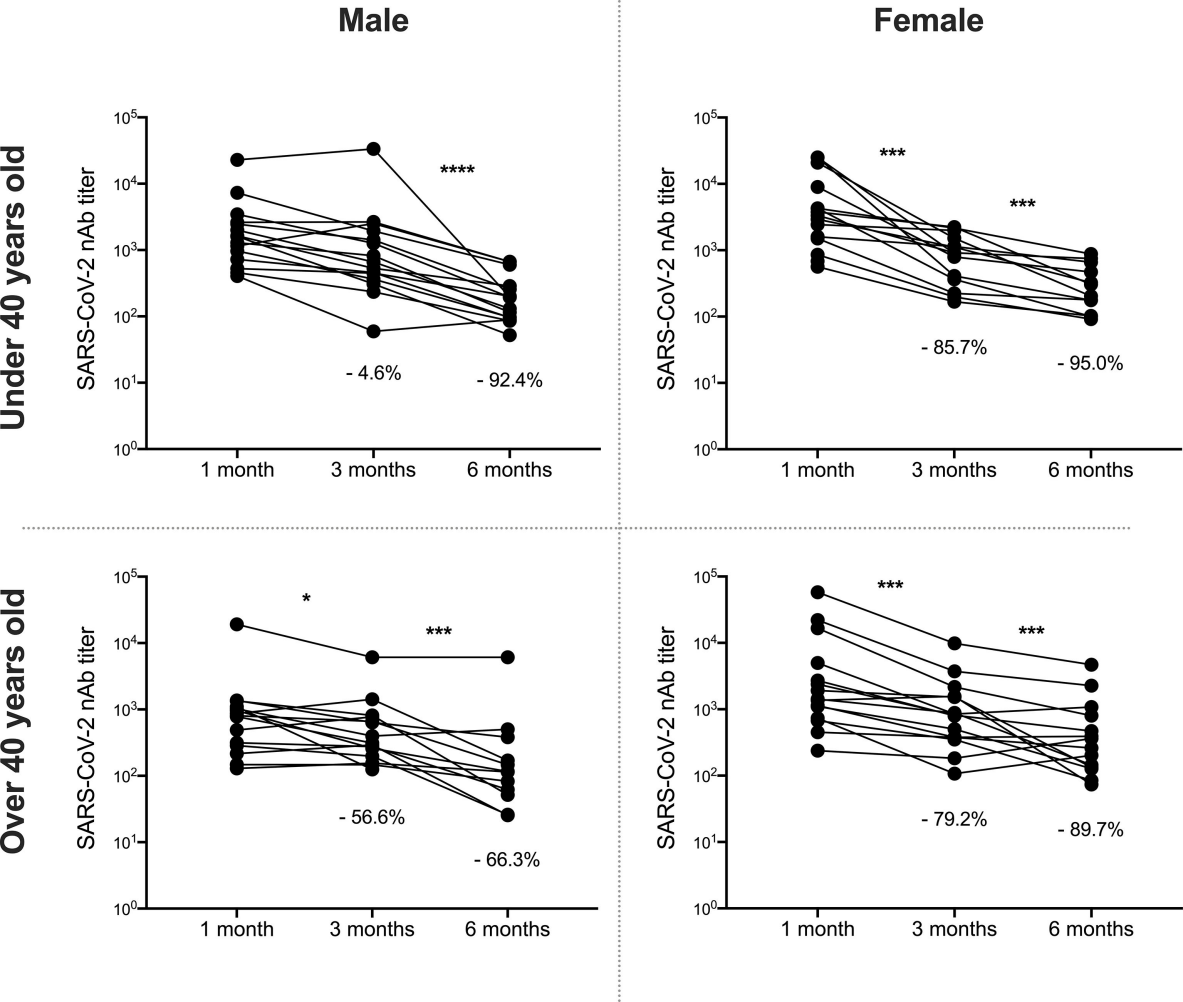
Decrease nAbs against B.1 lineage over time, according to age and gender. Neutralizing antibody (nAbs) titers against B.1 virus are shown between individuals one month and three months after being fully vaccinated and compared according to age (over or under 40 years) and sex. The statistical analysis was performed using Wilcoxon’s test. *p<0.05, ***p<0.001, ****p<0.0001.

The kinetics of the PRNTi against B.1 lineage of SARS-CoV-2 were not different according to gender since both males and females had a marked drop at 180 days post-vaccination (**Figure 4**). Although women reached high neutralizing titers at day 30, after 90- and 180-days post-vaccination, they showed a marked drop, both older and younger than 40 years old (95.0% and 89.7%, respectively at 180 days) (**Figure 4**). On the other hand, in males, a significant reduction in neutralizing activity was observed after three months, only in those older than 40 years old, since the younger males only showed a significant reduction at six months (**Figure 4**).

As PRNTi against all variants was affected, we analyzed these results in independent groups according to age and gender. After six months, neutralizing titers showed a drastic reduction against Gamma, Alpha and Delta variants in all groups of donors. Notably, the reduction was observed for the Delta variant in all groups analyzed in both three- and six-month follow-up. For the Mu variant, the reduction in nAbs titers was differentially modulated according to age and gender (**Figure S1**).

## Discussion

Ever since the design of currently rolled-out vaccines against SARS-CoV-2, evidence has shown their remarkable effectiveness in preventing infection, particularly severe disease and death related to COVID-19. Yet, growing evidence has focused on the potential harm of variants against established immunity, varying from *in silico* to observational population analyses and even nationwide studies (4, 14, 15). Indeed, our results lead to a similar conclusion. When serum neutralizing activity was first evaluated at one-month post-vaccination, all individuals displayed a peak of neutralizing response against B.1 lineage SARS-CoV-2, as expected. Of note, mRNA in the BNT162b2 vaccine encodes a nearly identical Spike protein to the reference SARS-CoV-2 genome, Wuhan-Hu-1 (16), which might explain this vaccine’s outstanding performance against relatively ancestral lineages such as B.1 virus, evaluated in this study. From this perspective, neutralizing response reached peak levels and behaved consistently in all groups, although a lower response was seen among older individuals. Furthermore, among participants in our study, females displayed a fairly greater PNRTi than males, in concordance with previous studies with different vaccines (17).

However, once we evaluated cross-reactivity against other variants, titers fell consistently among groups. VOCs Gamma, Alpha, and Delta, many of which exhibit mutations related to immune escape such as E484K and N501Y, have been extensively documented to display an increased capacity for evading antibody responses (7). Yet, one of our most noticeable findings is that Mu, first detected in January 2021 in Colombia (18), and the WHO listed as a VOI due to its ability to increase community transmission and higher prevalence in an area, features the escape mutations E484K, N501Y, and K417N. This last mutation has been detected in Delta plus (AY.4.2) and is also related to immune evasion (19). The results obtained at 30 days post-vaccination showcase a reduced response against all variants assessed, when compared to ancestral B.1, especially for Mu which displays a significantly higher molecular divergence. Hence, our results suggest that as variants genetically drift away from the reference genome, vaccine efficacy might be compromised.

Furthermore, our evidence demonstrates that the neutralizing response against SARS-CoV-2 and its variants wanes over time in vaccinated individuals. Previous work from several authors goes in line with this as well, both in convalescent and vaccinated individuals (20, 21). We observed a dramatic decrease in PNRTi titers against B.1 lineage of SARS-CoV-2 at three- and six months post-vaccination. The neutralizing response has been the predominant immunological marker for protection against SARS-CoV-2. Some studies estimate that the 50% protective neutralization levels, as a correlate of protection for severe disease, would be between 1:10 and 1:30, even though it could be as high as 1:200 (13, 22). Hence, the resulting antibody titers against B.1 at 3 and 6 months (at around 1:1660 and 1:505) would still be sufficient.

In the case of variants, the decay of humoral response is different. VOCs such as Alpha and Gamma would not likely translate into a threat for vaccinated individuals since the rates of decrease in antibody levels are not as strong (both remain over 1:1000 for the most part) as has been shown in other studies (23). For Delta, however, the decay is more remarkable, and the resulting mean nAbs titers approach 1:500 at three months, a decrease also observed by other authors (24). The most alarming data are those from Mu, as titers cross the 1:200 threshold at three months post-vaccination. Even though correlates of protection in variants may behave differently to those in B.1 lineage of SARS-CoV-2, it is safe to say that variants Delta and Mu have an outstanding ability to evade antibody responses in BNT162b2-vaccinated individuals and that immunity against them wanes faster than against other, less-circulating variants.

The results obtained from our study indicate that the humoral immune response induced by BNT162b2 holds less robustly against some of the VOCs and VOI that have circulated in Colombia, particularly against Delta and Mu, this last, the most unsuccessfully neutralized lineage assessed and the variant with the highest waning of them all (25–27). Consistent with our findings, Álvarez-Díaz et al. found that serum neutralizing activity from individuals vaccinated with BNT162b2 decreased by 75.7- and 17.7-fold against Mu, with respect to B.1.111 lineage and Gamma variant, respectively (26). In addition, Uriu et al. showed that the Mu variant was 9.1 as resistant as the ancestral lineage and 1.5 times as resistant to neutralization by serum from individuals vaccinated as the Beta variant (25). Further, Tada et al. reported that Mu and C.1.2 variants were more resistant to neutralization by BNT162b2 vaccination (6.8- and 7.3-fold decrease in titer, respectively, compared with D614G strain) (27). Based on the exposed view in Colombia and other countries (25–27), it should be considered that Mu be classified as a VOC since vaccines’ effectiveness has decreased to a certain extent.

On the other hand, the decrease in antibody titers after vaccination is common for different vaccines. Some authors have reported a more significant decrease in antibodies against influenza B strains than A strains after six months of vaccination (28). Others have reported that antibody levels and avidity decrease between 8% and 23% after six months to 20 years of vaccination against measles, mumps, and rubella (29). However, in the context of vaccination for SARS-CoV-2, our study shows that the decrease in the nAbs is more pronounced. These results suggest that time would render a substantial amount of the population susceptible to acquiring infection, especially as variants continue to emerge with increasing genetic divergence. This holds especially true for the elderly, considering that older individuals mount lower titers against all variants, and the kinetics of nAbs have shown an age dependency (30, 31). Considering that older individuals have a higher risk of suffering complications related to COVID-19, they must be protected from an ever-growing body of SARS-CoV-2 variants.

As shown herein and by other authors, the neutralizing response against mRNA vaccines decreases over time but is also commonly affected by mutations in SARS-CoV-2 variants (26, 32). This phenomenon occurs with other types of vaccines. For example, the humoral immune response triggered by adenoviral vaccines is less efficient in variants neutralization than mRNA vaccines (33, 34), and the sensitivity to neutralizing antibodies also decreases.

Our results showed a clear correlation between age, gender, and rate of decay in the post-vaccination immune response, remarkable at the peak neutralizing response and reduced near the end of the follow-up. Recently, other authors have reported that the humoral response to the BNT162b2 vaccine is affected by gender and age in studies carried out with ancestral lineages such as B.1 (31, 35, 36). The critical issue we observed in our study is that neutralization of variants also showed a differential reduction, especially in males over 40 years old. Clearly, males, especially older individuals, showed a significant decrease in nAbs titers against Alpha, Gamma, Delta, and Mu, aligning with other reports’ conclusions (31). Furthermore, even though our group of older individuals did not report having several comorbidities, this population tends to accumulate more risk factors, and antibody titers tend to decline sharply (37).

An advantage of our design is the spectrum of epidemiologically, and immunologically relevant variants studied. This diverse sample allowed for a proper and direct comparison between lineages that previously depended on normalization or data extrapolation. Moreover, we assessed neutralization through PRNT at serial dilutions, which is regarded as the gold standard for measuring antibody levels for many viruses, including SARS-CoV-2. However, the study has limitations: First, the overall sample size is restricted. Second, as this is an observational study, several variables were not precisely controlled. Third, it is possible that participants could have been infected asymptomatically, which could present as a confounding variable. Despite classical vaccinology being focused on the induction of antibodies (mainly neutralizing), cellular-mediated immunity is currently being recognized as an important aim of vaccination, which needs to be assessed to understand the overall adaptive immune response. This cohort, as well as the cellular immunity, will be assessed in future studies.

In conclusion, our study provides evidence of the humoral response kinetics in a heterogeneous population followed for six months. The neutralizing capacity was evaluated against widely distributed variants in the world. Since the pandemic continues to be a public health challenge to face, it is imperative to have protection correlates after vaccination and to have extensive information on the immune response generated by these vaccines against worrisome variants, especially when it comes to malleable and quickly produced platforms. Another study evaluating the cell-mediated immunity to SARS-CoV-2 vaccination will be developed to better elucidate the immunologic effects of SARS-CoV-2 vaccines. In addition, the evaluation of immune parameters induced by SARS-CoV-2 infection in infected individuals with different clinical outcomes will be reported in future publications.

## Data Availability Statement

The original contributions presented in the study are included in the article/**Supplementary Material**. Further inquiries can be directed to the corresponding author.

## Ethics Statement

The studies involving human participants were reviewed and approved by Ethics Committee of the Universidad de Antioquia (Acta 006/2021). The patients/participants provided their written informed consent to participate in this study.

## Author Contributions

TL and MC-M, formal analysis and writing – original draft. LF-A and MZ-C, investigation and formal analysis. NT, formal analysis, visualization, and writing - original draft. MR, conceptualization, project administration, writing - review and editing. JH, formal analysis, software, conceptualization, writing - original draft, and supervision. All authors contributed to the article and approved the submitted version.

## Funding

This study was supported by *Universidad de Antioquia, Universidad Cooperativa de Colombia* and *Corporación Universitaria Remington*. The funders had no role in the study’s design, data collection, and analysis, the decision to publish, or the preparation of the manuscript.

## Conflict of Interest

The authors declare that the research was conducted in the absence of any commercial or financial relationships that could be construed as a potential conflict of interest.

## Publisher’s Note

All claims expressed in this article are solely those of the authors and do not necessarily represent those of their affiliated organizations, or those of the publisher, the editors and the reviewers. Any product that may be evaluated in this article, or claim that may be made by its manufacturer, is not guaranteed or endorsed by the publisher.

## Supplementary Material

Supplementary Figure 1 Decreased nAbs overtime against different variants of SARS-CoV-2, according to age and gender. Neutralizing antibody (nAb) titers against Gamma, Alpha, Delta, and Mu are shown between individuals at one, three, and six months after being fully vaccinated and compared according to age (over or under 40 years) and sex. The statistical analysis was performed using Wilcoxon's test. *p<0.05. **p<0.01. ***p<0.001.

## References

[1] Johns Hopkins University & Medicine. COVID-19 Map - Johns Hopkins Coronavirus Resource Center. Johns Hopkins Coronavirus Resour Cent, Baltimore, Maryland (2020).

[2] Polack FP Thomas SJ Kitchin N Absalon J Gurtman A Lockhart S. Safety and Efficacy of the BNT162b2 mRNA Covid-19 Vaccine. N Engl J Med (2020) 383:2603–15. doi: 10.1056/NEJMoa2034577 PMC7745181 33301246

[3] Baden LR El Sahly HM Essink B Kotloff K Frey S Novak R. Efficacy and Safety of the mRNA-1273 SARS-CoV-2 Vaccine. N Engl J Med (2020) 384:403–16. doi: 10.1056/NEJMoa2035389 PMC7787219 33378609

[4] Tartof SY Slezak JM Fischer H Hong V Ackerson BK Ranasinghe ON. Effectiveness of mRNA BNT162b2 COVID-19 Vaccine Up to 6 Months in a Large Integrated Health System in the USA: A Retrospective Cohort Study. Lancet (London England) (2021) 398:1407–16. doi: 10.1016/S0140-6736(21)02183-8 PMC8489881 34619098

[5] World Health Organization. Tracking SARS-CoV-2 Variants. WHO, Ginebra, Swizertland, (2021). Available at: https://www.who.int/en/activities/tracking-SARS-Co.

[6] Romano M Ruggiero A Squeglia F Maga G Berisio R. A Structural View of SARS-CoV-2 RNA Replication Machinery: RNA Synthesis, Proofreading and Final Capping. Cells (2020) 9(5):1267. doi: 10.3390/cells9051267 PMC7291026 32443810

[7] Harvey WT Carabelli AM Jackson B Gupta RK Thomson EC Harrison EM. SARS-CoV-2 Variants, Spike Mutations and Immune Escape. Nat Rev Microbiol (2021) 19:409–24. doi: 10.1038/s41579-021-00573-0 PMC8167834 34075212

[8] Lopez Bernal J Andrews N Gower C Gallagher E Simmons R Thelwall S. Effectiveness of Covid-19 Vaccines Against the B.1.617.2 (Delta) Variant. N Engl J Med (2021) 385:585–94. doi: 10.1056/NEJMoa2108891 PMC8314739 34289274

[9] Folegatti PM Ewer KJ Aley PK Angus B Becker S Belij-Rammerstorfer S. Safety and Immunogenicity of the ChAdOx1 Ncov-19 Vaccine Against SARS-CoV-2: A Preliminary Report of a Phase 1/2, Single-Blind, Randomised Controlled Trial. Lancet (2020) 396:467–78. doi: 10.1016/S0140-6736(20)31604-4 PMC7445431 32702298

[10] Chvatal-Medina M Mendez-Cortina Y Patiño PJ Velilla PA Rugeles MT. Antibody Responses in COVID-19: A Review. Front Immunol (2021) 12:633184. doi: 10.3389/fimmu.2021.633184 33936045 PMC8081880

[11] Altawalah H. Antibody Responses to Natural SARS-CoV-2 Infection or After COVID-19 Vaccination. Vaccines (2021) 9(8):910. doi: 10.3390/vaccines9080910 34452035 PMC8402626

[12] Poonia B Kottilil S. Immune Correlates of COVID-19 Control. Front Immunol (2020) 11:569611. doi: 10.3389/fimmu.2020.569611 33133083 PMC7550526

[13] Addetia A Crawford KHD Dingens A Zhu H Roychoudhury P Huang M-L. Neutralizing Antibodies Correlate With Protection From SARS-CoV-2 in Humans During a Fishery Vessel Outbreak With a High Attack Rate. J Clin Microbiol (2020) 58(11):2107–20. doi: 10.1128/JCM.02107-20 PMC7587101 32826322

[14] Villoutreix BO Calvez V Marcelin AG Khatib AM. In Silico Investigation of the New UK (B.1.1.7) and South African (501y.V2) SARS-CoV-2 Variants With a Focus at the Ace2–Spike Rbd Interface. Int J Mol Sci (2021) 22:1–13. doi: 10.3390/ijms22041695 PMC7915722 33567580

[15] Charmet T Schaeffer L Grant R Galmiche S Chény O Von Platen C. Impact of Original, B.1.1.7 and B.1.351/P.1 SARS-CoV-2 Lineages on Vaccine Effectiveness of Two Doses of COVID-19 mRNA Vaccines: Results From a Nationwide Case-Control Study in France. Lancet Reg Heal - Eur (2021) 8:100171. doi: 10.1016/j.lanepe.2021.100171 PMC8277121 34278372

[16] Xia X. Detailed Dissection and Critical Evaluation of the Pfizer/BioNTech and Moderna mRNA Vaccines. Vaccines (2021) 9(7):734. doi: 10.3390/vaccines9070734 34358150 PMC8310186

[17] Fink AL Klein SL. The Evolution of Greater Humoral Immunity in Females Than Males: Implications for Vaccine Efficacy. Curr Opin Physiol (2018) 6:16–20. doi: 10.1016/j.cophys.2018.03.010 30320243 PMC6181235

[18] Instituto Nacional de Salud. Noticias coronavirus-genoma. Inst Nac Salud, Bogotá, Colombia (2021).

[19] Public Health England. SARS-CoV-2 Variants of Concern and Variants Under Investigation in England. Sage (2021) 01:1–50. Available at: https://www.gov.uk/government/publications/investigation-of-sars-cov-2-variants-technical-briefings.

[20] Goldberg Y Mandel M Bar-On YM Bodenheimer O Freedman L Haas EJ. Waning Immunity After the BNT162b2 Vaccine in Israel. N Engl J Med (2021) 385:e85. doi: 10.1056/NEJMoa2114228 34706170 PMC8609604

[21] Xia W Li M Wang Y Kazis LE Berlo K Melikechi N. Longitudinal Analysis of Antibody Decay in Convalescent COVID-19 Patients. Sci Rep (2021) 11:16796. doi: 10.1038/s41598-021-96171-4 34408200 PMC8373894

[22] Khoury DS Cromer D Reynaldi A Schlub TE Wheatley AK Juno JA. Neutralizing Antibody Levels are Highly Predictive of Immune Protection From Symptomatic SARS-CoV-2 Infection. Nat Med (2021) 27:1205–11. doi: 10.1038/s41591-021-01377-8 34002089

[23] Mileto D Fenizia C Cutrera M Gagliardi G Gigantiello A De Silvestri A. SARS-CoV-2 mRNA Vaccine BNT162b2 Triggers a Consistent Cross-Variant Humoral and Cellular Response. Emerg Microbes Infect (2021) 10:2235–43. doi: 10.1080/22221751.2021.2004866 PMC8648019 34749573

[24] Davis C Logan N Tyson G Orton R Harvey WT Perkins JS. Reduced Neutralisation of the Delta (B.1.617.2) SARS-CoV-2 Variant of Concern Following Vaccination. PLoS Pathog (2021) 17:e1010022. doi: 10.1371/journal.ppat.1010022 34855916 PMC8639073

[25] Uriu K Kimura I Shirakawa K Takaori-Kondo A Nakada T-A Kaneda A. Neutralization of the SARS-CoV-2 Mu Variant by Convalescent and Vaccine Serum. N Engl J Med (2021) 385:2397–9. doi: 10.1056/NEJMc2114706 PMC8609602 34731554

[26] Álvarez-Díaz DA Muñoz AL Tavera-Rodríguez P Herrera-Sepúlveda MT Ruiz-Moreno HA Laiton-Donato K. Low Neutralizing Antibody Titers Against the Mu Variant of SARS-CoV-2 in 31 BNT162b2 Vaccinated Individuals in Colombia. Vaccines (2022) 10(2):180. doi: 10.3390/vaccines10020180 35214639 PMC8876570

[27] Tada T Zhou H Dcosta BM Samanovic MI Cornelius A Herati RS. High-Titer Neutralization of Mu and C.1.2 SARS-CoV-2 Variants by Vaccine-Elicited Antibodies of Previously Infected Individuals. Cell Rep (2022) 38:110237. doi: 10.1016/j.celrep.2021.110237 34982967 PMC8687746

[28] Lee JH Cho HK Kim KH Lee J Kim YJ Eun BW. Evaluation of Waning Immunity at 6 Months After Both Trivalent and Quadrivalent Influenza Vaccination in Korean Children Aged 6-35 Months. J Korean Med Sci (2019) 34:e279. doi: 10.3346/jkms.2019.34.e279 31779056 PMC6882944

[29] Kontio M Jokinen S Paunio M Peltola H Davidkin I. Waning Antibody Levels and Avidity: Implications for MMR Vaccine-Induced Protection. J Infect Dis (2012) 206:1542–8. doi: 10.1093/infdis/jis568 22966129

[30] Müller L Andrée M Moskorz W Drexler I Walotka L Grothmann R. Age-Dependent Immune Response to the Biontech/Pfizer BNT162b2 Coronavirus Disease 2019 Vaccination. Clin Infect Dis (2021) 73:2065–72. doi: 10.1093/cid/ciab381 PMC8135422 33906236

[31] Bates TA Leier HC Lyski ZL Goodman JR Curlin ME Messer WB. Age-Dependent Neutralization of SARS-CoV-2 and P.1 Variant by Vaccine Immune Serum Samples. JAMA (2021) 326:868–9. doi: 10.1001/jama.2021.11656 PMC8295896 34287620

[32] Noori M Nejadghaderi SA Arshi S Carson-Chahhoud K Ansarin K Kolahi AA. Potency of BNT162b2 and mRNA-1273 Vaccine-Induced Neutralizing Antibodies Against Severe Acute Respiratory Syndrome-CoV-2 Variants of Concern: A Systematic Review of *In Vitro* Studies. Rev Med Virol (2021) 32(2):1–23. doi: 10.1002/rmv.2277 PMC8420542 34286893

[33] Terpos E Trougakos IP Karalis V Ntanasis-Stathopoulos I Sklirou AD Bagratuni T. Comparison of Neutralizing Antibody Responses Against SARS-CoV-2 in Healthy Volunteers Who Received the BNT162b2 mRNA or the AZD1222 Vaccine: Should the Second AZD1222 Vaccine Dose be Given Earlier? Am J Hematol (2021) 96:E321–4. doi: 10.1002/ajh.26248 PMC8212114 34028867

[34] Tada T Zhou H Samanovic MI Dcosta BM Cornelius A Herati RS. Neutralization of SARS-CoV-2 Variants by mRNA and Adenoviral Vector Vaccine-Elicited Antibodies. Front. Immunol (2022) 13:797589. doi: 10.3389/fimmu.2022.797589 PMC8957851 35350781

[35] Lustig Y Sapir E Regev-Yochay G Cohen C Fluss R Olmer L. BNT162b2 COVID-19 Vaccine and Correlates of Humoral Immune Responses and Dynamics: A Prospective, Single-Centre, Longitudinal Cohort Study in Healthcare Workers. Lancet Respir Med (2021) 9:999–1009. doi: 10.1016/S2213-2600(21)00220-4 34224675 PMC8253545

[36] Levin EG Lustig Y Cohen C Fluss R Indenbaum V Amit S. Waning Immune Humoral Response to BNT162b2 Covid-19 Vaccine Over 6 Months. N Engl J Med (2021) 385:e84. doi: 10.1056/NEJMoa2114583 34614326 PMC8522797

[37] Michos A Tatsi E-B Filippatos F Dellis C Koukou D Efthymiou V. Association of Total and Neutralizing SARS-CoV-2 Spike -Receptor Binding Domain Antibodies With Epidemiological and Clinical Characteristics After Immunization With the 1st and 2nd Doses of the BNT162b2 Vaccine. Vaccine (2021) 39:5963–7. doi: 10.1016/j.vaccine.2021.07.067 PMC8302834 34400017

